# Selective divergence between Grokipedia and Wikipedia articles

**DOI:** 10.1073/pnas.2603294123

**Published:** 2026-05-15

**Authors:** Saeedeh Mohammadi, Taha Yasseri

**Affiliations:** ^a^Centre for Sociology of Humans and Machines, Trinity College Dublin and Technological University Dublin, Dublin D02 PN40, Ireland; ^b^School of Mathematics and Statistics, University College Dublin, Dublin D04 V1W8, Ireland

**Keywords:** Grokipedia, Wikipedia, political bias, large language models, content analysis

## Abstract

The launch of Grokipedia, an AI-generated encyclopedia developed by xAI, was presented as a response to perceived ideological and structural biases in Wikipedia, with the goal of producing more “truthful” entries using the Grok large language model. However, whether such an AI-driven alternative can systematically correct the biases and limitations of human-edited platforms remains unclear. Here we conduct a large-scale computational comparison of 17,790 matched article pairs drawn from the 20,000 most-edited English Wikipedia pages. We find that Grokipedia pages are longer, more syntactically complex, and contain fewer references per word. Similarity measures across the two platforms reveal a bimodal structure: many Grokipedia articles closely resemble their Wikipedia counterparts, while a considerable subset diverges. Political bias differences emerge primarily within the divergent subset, where Grokipedia shows a relative rightward shift in the ideological orientation of frequently cited news media sources, particularly in articles related to religion and history. These patterns indicate selective, topic-specific divergence rather than a uniform debiasing of Wikipedia content. More broadly, AI-generated encyclopedias may depart from established editorial norms by favoring narrative expansion over citation-based verification, raising questions about transparency, provenance, and the governance of knowledge in automated information systems.

Online encyclopedias are central to public knowledge. Wikipedia remains the paradigmatic example: a volunteer-edited project governed by principles such as neutrality and verifiability. Despite its success, it has long faced questions about bias, reliability, and systemic underrepresentation (1, 2).

In late 2025, xAI introduced *Grokipedia*, an AI-generated encyclopedia positioned as an alternative to Wikipedia. According to xAI, it aims to “purge out the propaganda” and provide “truthful” entries, with content generated and internally “fact-checked” by the Grok language model rather than curated by human editors. At launch, Grokipedia reportedly contained roughly 800,000 to 900,000 entries, used a suggest-edit rather than a direct-edit workflow, and offered limited information about the data sources, licensing arrangements, and technical infrastructure used to generate content. Early commentary raised concerns that many pages were copied or closely adapted from Wikipedia (3).

Beyond this specific platform, Grokipedia represents a broader shift toward AI-generated knowledge systems. Large language models (LLMs) are increasingly used to produce content that may shape public access to information and the training data of future models. Understanding how these systems differ from collaborative platforms such as Wikipedia is therefore an important question for the governance and reliability of digital knowledge infrastructures.

This launch revived a longstanding question: is Wikipedia biased, and can an AI-generated encyclopedia do better? Prior work documents multiple forms of bias in Wikipedia, including early evidence of modest left-leaning political slant that attenuated over time (1), topical and coverage imbalances (2), framing asymmetries across ideology and gender (4, 5), and well-documented editorial conflicts (6).

Work on LLMs shows systematic political and cultural biases that vary across architectures and prompts (7, 8). LLMs also exhibit framing-sensitive and intrinsic political biases (9). Comparative studies suggest that Grok is less prone than many models to fabricating references, although citation inaccuracies remain common (10, 11).

Early analyses of Grokipedia discuss systematic differences from Wikipedia in citation patterns, article length, and political framing, and suggest that this divergence is concentrated in politically and culturally sensitive domains (12–14). Our analysis complements this literature by providing a large-scale comparison and examining content similarity alongside shifts in ideological orientation.

We compare Grokipedia entries matched to the 20,000 most-edited English Wikipedia articles. We examine differences in textual similarity, structural features, and the political orientation of cited news media sources, and assess how these vary across topical domains.

## Results

To assess how closely Grokipedia mirrors Wikipedia, we computed lexical, n-gram, semantic, and stylistic similarity metrics for each matched article pair (*SI Appendix*). Similarity scores are bimodal across all measures. We therefore derived a combined similarity score using principal components analysis (PCA). The first component explains 94% of the variation and is itself distinctly bimodal, indicating two substantive groups of article pairs: one highly similar (34% of pairs) and one more divergent (66% of pairs).

Across the corpus, Grokipedia articles tend to be longer than their Wikipedia counterparts and exhibit more complex, less accessible prose (Table 1). On average, Grokipedia entries show greater reading difficulty and slightly greater lexical diversity, while Wikipedia articles contain substantially more references and hyperlinks per 1,000 words. These differences are driven primarily by highly dissimilar article pairs, in which Grokipedia pages are longer and more complex, while relying on fewer references. Among highly similar pairs, the two platforms are much closer in length, readability, and lexical diversity.

**Table 1.** Comparison of structural and linguistic properties between Wikipedia and Grokipedia articles

	All pairs (17,790)		Highly similar pairs (6,089)		Highly different pairs (11,701)		
Metric	Wikipedia	Grokipedia	Wikipedia	Grokipedia	Wikipedia	Grokipedia	*P*-value
Mean article length (words)	6,280 (3,828)	7,662 (3,662)	5,062 (3,296)	5,125 (3,005)	6,915 (3,932)	8,983 (3,256)	<0.001
Mean Flesch–Kincaid grade	10.7 (1.7)	14.5 (3.9)	10.2 (1.6)	10.0 (2.1)	10.9 (1.6)	16.9 (2.2)	<0.001
Mean type–token ratio	0.28 (0.06)	0.29 (0.05)	0.28 (0.07)	0.28 (0.07)	0.28 (0.06)	0.29 (0.04)	<0.001
Mean references per 1k words	35 (37)	20 (10)	34 (44)	21 (15)	35 (33)	21 (5)	<0.001
Mean hyperlinks per 1k words	357 (323)	24 (16)	383 (375)	30 (25)	343 (292)	21 (6)	<0.001

Values represent mean (SD). The *P*-values are calculated using two-tailed paired *t* tests. See *SI Appendix* for details.

Another dimension of article change concerns political leaning. We examined the sources cited in each article and assigned political bias scores using publicly available datasets (*SI Appendix*). These datasets primarily cover widely shared media domains; therefore, this measure captures differences in the ideological orientation of frequently cited media sources as a proxy for differences in the political leaning of the articles. For each matched pair, the bias shift is defined as the difference between the average bias score of the Grokipedia article and that of its Wikipedia counterpart. Positive values indicate greater reliance on right-leaning sources in Grokipedia, whereas negative values indicate a shift toward left-leaning sources.

**Fig. 1.**
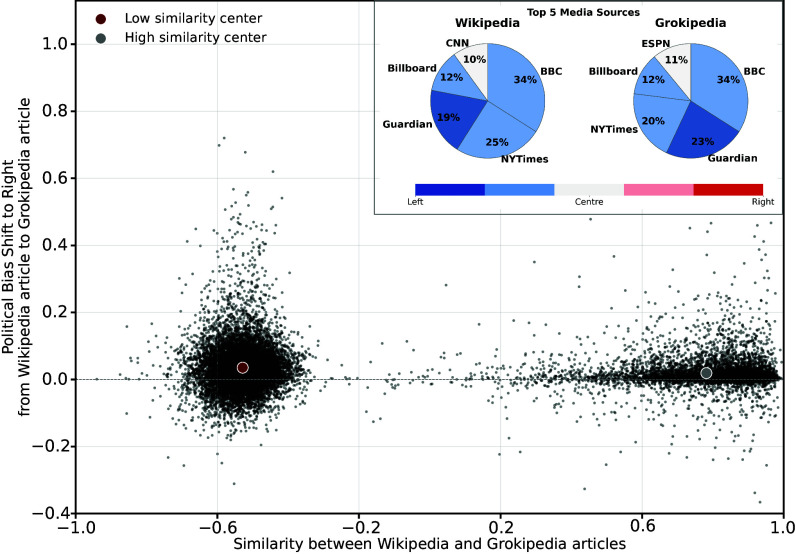
Bias shift vs. article similarity. Each point represents an article pair. The *x*-axis shows the combined similarity score between the Wikipedia and Grokipedia versions, and the *y*-axis shows the political bias shift (Grokipedia–Wikipedia). Positive values indicate a shift toward right-leaning sources, while negative values indicate a shift toward left-leaning sources. The markers denote the centers of mass for the low- and high-similarity groups. *Inset*: Pie charts show the top five news domains cited in Wikipedia (*Left*) and Grokipedia (*Right*) among articles with low combined similarity scores, weighted by citation frequency. Slice areas represent relative contribution, and colors encode political bias.

Fig. 1 plots the bias shift for each article pair against its combined similarity score. Although the average shift among low-similarity articles is rightward, many articles exhibit leftward shifts, reflecting heterogeneous source adjustment. Examples are provided in the Dataset S1. The *Inset* displays the five most frequently cited news sources within the low-similarity group for each platform: Grokipedia articles exhibit a relative rightward shift in sourcing, yet remain predominantly left-leaning.

**Fig. 2.**
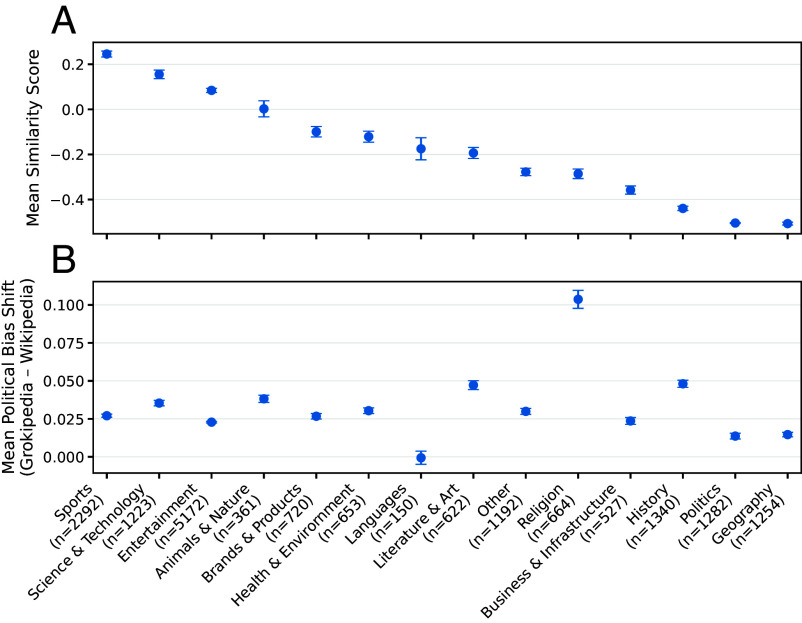
Topic-level comparison of similarity and political bias shift between Wikipedia and Grokipedia articles across the whole sample. (*A*) Mean combined similarity scores between Wikipedia and Grokipedia articles within each topic, with SE bars. (*B*) Mean political bias shift (Grokipedia–Wikipedia) for articles in each topic, indicating whether Grokipedia content tends to lean more left or right relative to Wikipedia. Error bars indicate the SE for each topic.

Because the articles span a wide range of subjects, we categorized them into 14 topical domains, enabling comparisons of similarity and bias shift across the full sample. Fig. 2 shows that the largest cross-platform differences occur in geography, politics, history, business and infrastructure, and religion, while the strongest rightward shifts in source bias appear in religion, history, and literature and art.

## Discussion

Structurally, Grokipedia articles are generally longer and exhibit more syntactically complex, less accessible prose, while containing fewer references per word. This pattern contrasts with prior findings on Wikipedia’s language dynamics (15), in which human editors often simplified language without altering the vocabulary. In Grokipedia, by contrast, sentence length and readability grades tend to increase, suggesting expansion of narrative structure rather than simplification. The platform also lacks internal links, limiting readers’ ability to navigate, contextualize, and explore related material.

Similarity measures reveal a bimodal structure: many Grokipedia pages closely resemble their Wikipedia counterparts, while a substantial subset diverges in content and structure. This pattern indicates a hybrid authorship process in which some articles closely replicate Wikipedia, whereas others are more extensively rewritten. Divergence in sourcing follows the same logic: while highly similar articles show little change, more extensively revised pages exhibit greater variance and, on average, a rightward shift in the orientation of cited news media sources, particularly in contested domains. Rather than reflecting a uniform correction of Wikipedia’s ideological profile, this produces a patchwork of localized adjustments, in which revised articles shift in their sourcing while minimally modified pages largely preserve Wikipedia’s original content.

This should be understood in the context of both methodological and substantive scope. Because available bias datasets primarily cover widely shared media domains and rely on audience-based measures derived from social media activity, our estimates capture the orientation of news media sources rather than the full spectrum of cited references or the ideological content of individual sources. At the same time, other work shows that Grokipedia and Wikipedia differ substantially in their broader composition of source types, including academic, governmental, civil-society, and user-generated sources (13). The present analysis, therefore, isolates one specific dimension of this broader epistemic shift, while other aspects of sourcing remain outside its scope.

Wikipedia’s openness renders bias visible and contestable through edits, disputes, and deliberation (16). Grokipedia replaces this process with opaque, automated authorship, embedding potential biases within model behavior rather than exposing them to scrutiny. Despite its stated corrective aim, Grokipedia functions less as an epistemic alternative than as an AI-mediated reconfiguration of Wikipedia.

As AI-generated reference content may itself be incorporated into future language models or used by other AI systems, these dynamics extend beyond a single platform. Without transparency, accountability, and sustained human oversight, derivative knowledge systems risk propagating existing biases into the epistemic foundations of automated information environments.

## Materials and Methods

From the 20,000 most-edited English-language Wikipedia articles, we identified 17,790 matches on Grokipedia and downloaded the corresponding HTML pages in November 2025. For each pair, we computed readability, lexical, and stylistic features and quantified cross-platform alignment using eight similarity measures spanning lexical, n-gram, semantic, and stylistic domains. Principal components analysis showed that these measures load strongly on a single latent dimension; we therefore use the first principal component as a combined similarity score. We estimated each article’s political bias by averaging the bias scores of its media sources and categorized articles into 14 topical domains using a multistage LLM classifier. Additional details are provided in *SI Appendix*.

## Supplementary Material

Appendix 01 (PDF)

## Data Availability

Data and code used for the analyses have been deposited in Zenodo (17).
