# Supplementary material for: Selective divergence between Grokipedia and Wikipedia articles
Source: Proc Natl Acad Sci U S A. 2026 May 15;123(20):e2603294123. doi: 10.1073/pnas.2603294123 (PMC13187788; doi:10.1073/pnas.2603294123)
Supplement: Supplementary file 1 — Appendix 01 (PDF) [file pnas.2603294123.sapp.pdf]

Supporting Information for

Selective divergence between Grokipedia and Wikipedia articles

Saeedeh Mohammadi and Taha Yasseri

Corresponding Author: Taha Yasseri
E-mail: taha.yasseri@tcd.ie

This PDF file includes:

Supporting text
SI References

Supporting Information Text

Data Collection. We analyzed the 20,000 most-edited English-language Wikipedia articles as of October 2025, identified via cumulative edit counts. To ensure that our comparison focused on substantive articles, we excluded all list-style pages as well as titles that were date- or year-like rather than topical. Specifically, we removed (a) pages with the title "List of Topic" (e.g., List of UFC champions), (b) calendar dates (e.g., March 11), (c) standalone years (1000–2099), (d) "Year in Topic" pages (e.g., 2003 in music), (e) "Deaths in Month Year" pages, and (f) standalone month names.

For each remaining title, we retrieved the corresponding entries from Wikipedia and Grokipedia, generating URLs of the form `https://en.wikipedia.org/wiki/<Title>` and `https://Grokipedia.com/page/<Title>`.

HTML pages were downloaded between November 5–11, 2025, using the `requests` library (Python 3.11), and then parsed using `BeautifulSoup4`. After host-aware text extraction (described below), we retained only article pairs in which both platforms produced at least 500 words of clean prose. Of the original 20,000 target titles, 17,790 matched pairs met these criteria and formed the final analytical sample.

Host-aware text and feature extraction. We implemented a host-aware parsing strategy tailored to each platform's HTML structure to maximize content fidelity.

For Wikipedia, extraction was restricted to the `#mw-content-text` container and limited to `<p>` and `<li>` elements. Infoboxes, metadata, tables, navboxes, and reference lists were removed prior to text collection.

For Grokipedia, we used a more adaptive extractor to identify the primary article-like container (`<main>`, `<article>`, or the largest `<div>`/`<section>`) and retained its narrative text while filtering out menus, advertisements, and high–link-density regions. Scripts, style elements, and sidebars were removed for both platforms.

We tokenized each cleaned article into sentences and words using `nltk`'s Punkt tokenizer.

From the extracted text of each article, we computed a set of stylistic and readability metrics.

- **Lexical diversity (type–token ratio):** Defined as the number of unique word types divided by the total number of word tokens. Higher values indicate greater vocabulary use and lower repetition in the text.
- **Flesch–Kincaid grade level:** A widely used readability formula that estimates the U.S. school grade level required to understand the text. It combines average sentence length and average syllables per word, with higher scores indicating more difficult material.

All features were computed independently for both platforms and labeled with prefixes `a_` (Grokipedia) and `b_` (Wikipedia).

Similarity Measures Between Platforms. To quantify alignment between articles on the two platforms, we computed four classes of similarity measures, each capturing a distinct dimension of textual resemblance:

- **Lexical similarity:** Lexical similarity was assessed using two complementary approaches. First, we computed cosine similarity between TF–IDF vectors constructed from 1–2 gram features with standard English stop-words removed. This measure captures similarity in word- and phrase-level vocabulary usage weighted by informativeness. Second, we calculated the unigram Jaccard index, defined as the ratio of the intersection to the union of unique word sets across platforms. This index measures the extent to which the two texts share the same vocabulary.
- **N-gram overlap:** To capture local phrase reuse and short-range structural similarity, we computed overlap coefficients for 1-, 2-, and 3-gram sequences. Each coefficient is defined as the size of the intersection of n-grams divided by the smaller of the two n-gram sets, providing a symmetric measure of shared phrase content independent of article length.
- **Semantic similarity:** We quantified semantic alignment using two embedding-based approaches. Cosine similarity between `SentenceTransformer` embeddings (`all-MiniLM-L6-v2`) measured the overall semantic closeness of the full articles in a dense representation space. In addition, we computed contextual similarity using `BERTScore` F1 on the first 50 sentences of each article, which evaluates token-level semantic correspondence using contextual embeddings (1, 2). Together, these metrics assess semantic similarity beyond surface-level wording.
- **Stylistic similarity:** Stylistic similarity was evaluated by computing the Manhattan distance between the two articles' stylistic feature vectors, which included sentence length, lexical diversity, readability metrics, and part-of-speech composition. The resulting distances were linearly transformed to a 0–1 scale, where higher values indicate greater similarity. This measure captures convergence in writing style independent of content.

To verify the extent to which these metrics reflected a single latent construct, we conducted a principal components analysis (PCA) on the full set of similarity measures. The first principal component accounted for the vast majority of total variance (94%), with all measures loading positively on the component (n–gram overlap 3: 0.528, n–gram overlap 2: 0.478, BERTScore F1: 0.458, lexical Jaccard unigram: 0.377, n–gram overlap 1: 0.295, lexical TF–IDF cosine: 0.198, semantic embedding cosine: 0.103, stylistic similarity: 0.071). This indicates that the metrics are largely colinear and capture a common underlying dimension of article similarity. Based on this result, we construct a unified combined similarity score defined as the standardized value of the first principal component and use it in all subsequent analyses. The standardized similarity score has a mean of −0.08 and an SD of 0.63.

Political Bias ratings. We extracted all outbound hyperlinks from each article and mapped their host domains to political-bias scores using a domain-level political-bias dataset. To consolidate hosts referring to the same outlet, we applied eTLD+1 normalization, supplemented by a brand key derived from the leftmost label. This procedure ensured consistent attribution across domain variants and subdomain structures, resulting in 186,678 and 219,814 unique domains for Wikipedia and Grokipedia, respectively. Bias scores correspond to the derived party leaning measure provided by Yang et al. (3). This political bias measure relies on audience-based estimates derived from social media sharing patterns, which approximate but do not directly measure the ideological content of individual sources. Scores were assigned when a value was available for the domain or any recognized brand variant (e.g., `bbc.com` and `bbc.co.uk`). Using this dataset, approximately 17% of unique cited domains could be matched to a political bias score (33,572 and 36,406 for Wikipedia and Grokipedia, respectively). Despite this relatively low domain-level coverage, the matched domains account for a large share of citation instances (typically 60–90%) in both encyclopedias, reflecting their higher citation frequency relative to unmatched domains. The full distribution of matched references per article is reported in (4). Across the entire sample, only 29 Grokipedia articles contained no matchable references. The distribution of matched domains per article is right-skewed, with most articles containing multiple matched sources.

To assess robustness, we replicated the analysis using an alternative bias dataset based on the News Media Bias and Factuality mapping by Sanchez et al. (5). Bias scores were converted to a symmetric numeric scale (-1 = left, 1 = right). This dataset provides substantially lower domain coverage (approximately 1% of referenced domains). Despite the large differences in coverage and dataset construction, the resulting bias patterns and overall conclusions remain consistent. See (4) for more details and analysis.

The bias score of each article is computed as the weighted average of the bias scores of its cited domains, where weights correspond to the number of times each domain appears in the page. As a robustness check, we also replicated the analysis without weighting domains by citation frequency. The resulting patterns remain qualitatively unchanged, although the magnitude of the shifts is slightly attenuated.

For each article pair, the bias shift was computed as the mention-weighted mean bias of matched domains in Grokipedia minus the corresponding mean in Wikipedia (positive values indicate a rightward shift; negative values indicate a leftward shift). Because the available bias datasets primarily cover news and widely shared media domains, this measure should be interpreted as capturing differences in the political orientation of frequently cited news-media-type sources rather than the full set of cited references.

Consistent with this interpretation, prior work shows that differences between Grokipedia and Wikipedia extend beyond news sources to broader shifts in source types, including academic, civil-society, and user-generated content (6). Our measure, therefore, captures one specific dimension of these differences—namely, the ideological orientation of frequently cited media domains—while other dimensions of sourcing remain outside its scope.

The centres of mass shown in Figure 1 were obtained using Gaussian kernel density estimation (KDE) on the article-level scatter points, computed separately for the low- and high-similarity partitions. Across both bias datasets, the results show a consistent pattern: for low-similarity articles, the centre shifts rightward relative to Wikipedia (Yang et al.: $y = 0.0353$; Sanchez et al.: $y = 0.0740$), whereas highly similar articles remain close to neutral (Yang et al.: $y = 0.0190$; Sanchez et al.: $y = -0.0119$). These shifts are modest in magnitude but consistent in direction across datasets, supporting the robustness of the observed pattern within the subset of matched sources.

For the pie charts in Figure 1, we restricted the analysis to the low-similarity subset (articles with a negative combined similarity score) and plotted the top 5 cumulative shares of referenced news outlets. For the pie charts, we focused exclusively on news sources, removing reference or utility hosts (e.g., `doi.org`, `archive.org`, `worldcat.org`), major platforms, and government or educational domains (`.gov`, `.edu`).

Categorisation. To assign each article to a topical domain, we implemented a multi-stage pipeline using a large language model (LLM). Article titles were processed in batches of up to 300 and submitted to the *gpt-5-nano-2025-08-07* model with explicit instructions to assign each title to exactly one topic and to avoid the use of "Other" unless no reasonable category applied. The model returned a JSON-formatted list of titles grouped by category. Outputs with formatting errors were automatically resubmitted, with up to five retries per batch. All valid outputs were merged into an initial table mapping each title to a category.

We used the following prompt:

```
You will receive a list of titles. Your task is to classify EACH title into EXACTLY ONE of the predefined
categories below.

IMPORTANT:
- Always choose the **closest** meaningful category.
- Use **"Other" only when NO reasonable category fits at all**.
- Try to avoid "Other" as much as possible.

Here are the STRICT categories you MUST choose from:
{categories_str}

Your output MUST be VALID JSON with this exact structure:
```

```
{{
  "History": [],
  "Politics_and_Political_Figures": [],
  "Geography_and_Nations_and_Cities": [],
  "Music_Movies_and_Entertainment_Celebrities": [],
  "Sports_and_Athletes_and_Teams": [],
  "Science_Technology_and_Knowledge": [],
  "Religion_and_Ideologies": [],
  "Brands_and_Products": [],
  "Languages": [],
  "Literature_and_Art": [],
  "Health_and_Environment": [],
  "Business_and_Infrastructure": [],
  "Animals_and_Nature": [],
  "Dates": [],
  "Other": []
}}

CLASSIFICATION RULES:
1. EVERY title must appear exactly once in the output.
2. DO NOT create new categories and DO NOT remove any.
3. **Avoid 'Other' unless absolutely impossible to classify elsewhere.**
4. When unsure, choose the category that is the **closest semantic match**.
5. Only use "Other" if the title genuinely does not relate to ANY listed category.
6. Output VALID JSON ONLY - no comments, no trailing commas.

Here are the titles:
```

Titles labeled as “Other” were isolated and resubmitted for reclassification in subsequent passes. This process was done four times. The results were then merged across passes. Titles that remained unclassified after all iterations were ultimately assigned to “Other”. This iterative integration procedure increased coverage and reduced the number of titles assigned to the “Other” category.

As a final step, we submitted each provisional category (batched in sets of 200 entries) to the *gpt-4.1-mini* model and instructed it to flag and propose revised labels for any titles that appeared misclassified. All suggested corrections were manually reviewed before inclusion in the final categorisation.

For this stage of the classification, we used the following prompt:

```
You are an expert content classifier. These titles were labeled under:
**{topic}**

Allowed categories:
{allowed_topics}

For each title, check if the topic assignment is correct.
If incorrect, recommend the correct topic.

Respond only in valid JSON:
[
  {{"title": "...", "assigned_topic": "...", "correct": true/false,
    "suggested_topic": "..." }}
]

Titles:
{batch}
```

Validation of topic classification. To assess the reliability of the automated classification procedure described above, we conducted several validation exercises. First, the entire LLM classification pipeline was executed six times independently, each using the same model and prompt. Agreement across these runs was high (Fleiss' $\kappa = 0.73$), indicating substantial consistency in the automated topic assignment. Second, we conducted a human validation exercise. A random sample of 200 article titles was independently annotated by six human coders using the same topical categories and instructions provided to the LLM. Inter-coder agreement among the human annotators was similarly high (Fleiss' $\kappa = 0.75$), indicating substantial agreement.

For both the automated and human annotations, a majority rule was used to determine the final topic assignment for each title. Comparison of the resulting human and machine classifications yields strong agreement (Cohen's $\kappa = 0.83$), suggesting that the automated classification procedure produces topic assignments closely aligned with human judgment. These results indicate that the LLM-based classification is both internally stable and externally consistent with human annotations. The majority-rule aggregation of the LLM runs was used as the final topic classification in the analysis.

Limitations. Our dataset is affected by several limitations. First, the dataset is drawn from Wikipedia's 20,000 most-edited English-language pages, of which 17,790 have counterparts on Grokipedia. This selection likely overrepresents high-profile and contentious topics—those most prone to editing disputes and ideological scrutiny (7). Second, the similarity metrics employed—lexical, semantic, structural, and stylistic—capture textual form and alignment but do not assess factual accuracy or ideological framing. As a result, hallucinated claims, selective omissions, or more subtle rhetorical biases remain beyond the scope of automated comparison. Third, the bias measure relies on the domain-level political orientation of cited sources, which is only an indirect proxy for the ideological framing of individual articles. Even with the broader bias dataset used in this study, only approximately 17% of cited domains could be matched to a bias score, because many references in encyclopedic content point to books and documents not included in media-bias datasets. Consequently, a substantial portion of citations fall outside the measurable sample. Fourth, both platforms are dynamic: Grokipedia and Wikipedia continue to evolve, and our analysis reflects only a snapshot of their content at the time of data collection. Finally, Grokipedia's underlying training data, retrieval mechanisms, and editorial interventions remain opaque, precluding full provenance auditing and limiting causal inference about the sources of observed bias. Despite these limitations, the large-scale comparative design, consistency of results across multiple metrics, and robustness checks provide a useful empirical characterization of emerging differences between AI-generated and human-edited encyclopedic knowledge systems.

Statistical and Comparative Analysis. All analyses were performed in Python 3.11 using `pandas`, `numpy`, and `scikit-learn`. We calculated the descriptive statistics (mean ± SD) for all features. We evaluated differences between Grokipedia and Wikipedia using paired t-tests. Spearman's rank correlation was computed among similarity metrics to assess interdependence and clustering. Visualizations, including histograms, correlation heatmaps, and mean ± SE summary plots, were generated with `matplotlib`.

Data, Materials, and Software Availability. Data and code used for the analyses are available at https://doi.org/10.5281/zenodo.19286583.

References

1. N Reimers, I Gurevych, Sentence-BERT: Sentence embeddings using siamese BERT-Networks in *Proceedings of the 2019 Conference on Empirical Methods in Natural Language Processing (EMNLP).* (Association for Computational Linguistics), pp. 3982–3992 (2019).
2. T Zhang, V Kishore, F Wu, KQ Weinberger, Y Artzi, BERTScore: Evaluating text generation with BERT in *International Conference on Learning Representations (ICLR).* (2020) https://openreview.net/forum?id=SkeHuCVFDr.
3. KC Yang, et al., Domaindemo: a dataset of domain-sharing activities among different demographic groups on twitter. *Sci. data* **12**, 1251 (2025).
4. T Yasseri, S Mohammadi, How similar are Grokipedia and Wikipedia? A multi-dimensional textual and structural comparison. *arXiv preprint arXiv:2510.26899* (2025) Accessed: 10/04/2026.
5. D Sánchez-Cortés, S Burdisso, E Villatoro-Tello, P Motlicek, Mapping the media landscape: predicting factual reporting and political bias through web interactions in *International Conference of the Cross-Language Evaluation Forum for European Languages.* (Springer), pp. 127–138 (2024).
6. A Mehdizadeh, M Hilbert, Epistemic substitution: How Grokipedia's AI-generated encyclopedia restructures authority. *arXiv preprint arXiv:2512.03337* (2025) Accessed: 31/03/2026.
7. T Yasseri, R Sumi, A Rung, A Kornai, J Kertész, Dynamics of conflicts in Wikipedia. *PLOS ONE* **7**, e38869 (2012).
